# Estimation of the carrier frequencies and proportions of potential patients by detecting causative gene variants associated with autosomal recessive bone dysplasia using a whole-genome reference panel of Japanese individuals

**DOI:** 10.1038/s41439-020-00133-7

**Published:** 2021-01-15

**Authors:** Shinichi Nagaoka, Yumi Yamaguchi-Kabata, Naomi Shiga, Masahito Tachibana, Jun Yasuda, Shu Tadaka, Gen Tamiya, Nobuo Fuse, Kengo Kinoshita, Shigeo Kure, Jun Murotsuki, Masayuki Yamamoto, Nobuo Yaegashi, Junichi Sugawara

**Affiliations:** 1 Graduate School of Medicine, Tohoku University, 2-1, Seiryo-machi, Aoba-ku, Sendai, 980-8575 Japan; 2 Tokyo Metropolitan Ohtsuka Hospital, 2-8-1, Minami-ohtsuka, Toshima-ku, Tokyo, 170-8476 Japan; 3 Tohoku Medical Megabank Organization, Tohoku University, 2-1, Seiryo-machi, Aoba-ku, Sendai, 980-8573 Japan; 4 Miyagi Cancer Center Research Institute, 47-1, Noda-yama, Medeshima-shiode, Natori, 981-1293 Japan; 5 Statistical Genetics Team, RIKEN Center for Advanced Intelligence Project, Nihonbashi 1-chome Mitui Building, 15th Floor, 1-4-1 Nihonbashi, Chuo-ku, Tokyo, 103-0027 Japan; 6 Graduate School of Information Sciences, Tohoku University, 6-3-09, Aza-aoba, Aramaki, Aoba-ku, Sendai, 980-8579 Japan; 7 Institute of Development, Aging and Cancer, Tohoku University, 4-1 Seiryo-machi, Aoba-ku, Sendai, 980-8575 Japan; 8 Advanced Research center for Innovations in Next-Generation Medicine, Tohoku University, 2-1 Seiryo-machi, Aoba-ku, Sendai, 980-9573 Japan

**Keywords:** Disease genetics, Genetics research

## Abstract

Bone dysplasias are a group of rare hereditary diseases, with up to 436 disease types. Perinatal diagnosis is clinically important for adequate personalized management and counseling. There are no reports focused on pathogenic variants of bone dysplasias in the general population. In this study, we focused on autosomal recessive bone dysplasias. We identified pathogenic variants using whole-genome reference panel data from 3552 Japanese individuals. For the first time, we were able to estimate the carrier frequencies and the proportions of potential patients. For autosomal recessive bone dysplasias, we detected 198 pathogenic variants of 54 causative genes. We estimated the variant carrier frequencies and the proportions of potential patients with variants associated with four clinically important bone dysplasias: osteogenesis imperfecta (OI), hypophosphatasia (HPP), asphyxiating thoracic dysplasia (ATD), and Ellis–van Creveld syndrome (EvC). The proportions of potential patients with OI, ATD, and EvC based on pathogenic variants classified as “pathogenic” and “likely pathogenic” by InterVar were closer to the reported incidence rates in Japanese subjects. Furthermore, the proportions of potential patients with HPP variants classified as “pathogenic” and “likely pathogenic” in InterVar and “pathogenic” in ClinVar were closer to the reported incidence rates. For bone dysplasia, the findings of this study will provide a better understanding of the variant types and frequencies in the Japanese general population, and should be useful for clinical diagnosis, genetic counseling, and personalized medicine.

## Bone dysplasia: Estimating carrier frequency with bioinformatics

A bioinformatics approach helps estimate carrier frequency of a rare inherited bone disease which causes abnormalities in skeletal shape and structure. Autosomal recessive bone dysplasias affect bone and cartilage development and result from inheriting two mutated genes, one from each parent. Junichi Sugawara, Tohoku University, Sendai, Japan, and colleagues used mutation databases and a bioinformatics tool for variant interpretation to detect 198 pathogenic variants in 54 genes associated with autosomal recessive bone dysplasia in a whole-genome reference panel of 3,552 general Japanese individuals (3.5KJPNv2). They then estimated the frequency of people in the sample carrying bone dysplasia mutations and the expected proportion in whom the disorder could manifest, which compared well with reported incidence rates in the general population. These findings could prove useful for calculating the risk of bone dysplasia in the future children of carrier parents.

## Introduction

Bone dysplasias are a group of inherited disorders caused by mutations in genes affecting the development and differentiation of bones and cartilage from the fetal stage, thereby resulting in abnormalities in skeletal shape and structure^1^. The clinical manifestations include a wide range of prognoses, from cases diagnosed during the fetal period to mild cases in adults that are difficult to diagnose^2,3^. The birth incidence of bone dysplasia is estimated to be ~1/5000 births^4,5^. The international classification of bone diseases involves a combination of single genes or multiple related genes, specific phenotypes, and radiological findings, and is updated every 4–5 years^6^. The classification from 2015 comprises 42 groups of bone dysplasias and 436 specific diseases^6^. Thus, the actual frequency of each disease is low. In addition, new genes and genetic variants are continually being reported due to developments in next-generation sequencing (NGS)^7,8^, and the number of causative genes and variants are expected to increase.

Determining the incidence of bone dysplasia and its diagnosis can be difficult. First, there have been few reports on the incidence of bone dysplasia in Western countries since the 1980s. The major reports were from Italy^4^ and South America^5^. In both reports, the incidence of each bone dysplasia was calculated as the sum of live births and stillbirths in a certain region or multiple countries over a period of several years. However, no survey of bone dysplasia incidence has been reported since that time. Furthermore, the trend in disease incidence according to racial differences has not been clarified. Second, there has been no nationwide survey of the incidence of bone dysplasia in Japan. Satoh et al.^9^ (article in Japanese) reported the prevalence of fetal bone dysplasia in obstetrics, which included children diagnosed at a single institution for 20 years and those registered at multiple institutions for 1 year. However, there was a large bias in the target population selection and the total number of patients was not reported. There have been no subsequent reports on the frequencies of bone dysplasia in Japan. As bone dysplasia is a rare hereditary disease, it is difficult to grasp the total number of cases via nationwide efforts and the target population setting is unclear even if the patients are registered at individual institutions. Third, new causative genes and variants predicting the diagnosis of bone dysplasia are continually being identified by NGS and confirmed for their utility in diagnosis^7,8^. NGS is useful for the diagnosis and discovery of causative genes and variants. However, estimated causative variants and carrier frequencies of genes related to bone dysplasia have not been reported in the general population. Carriers of disease-causing genetic variants may exist in the healthy general population. Therefore, using genomic information from the general population to investigate genetic variations and determine the frequency of mutation carriers and potential patients is helpful in understanding genetic epidemiology and applying it to perinatal care, genetic counseling, and personalized medicine.

We used 3.5KJPNv2^10,11^, a genome-wide allele-frequency reference panel, to estimate the carrier frequencies of variants associated with bone dysplasia in the Japanese population. In collaboration with the Tohoku Medical Megabank Organization and Iwate Tohoku Medical Megabank Organization, we conducted cohort studies using a biobank that integrates medical and genomic information from the general healthy population. Whole-genome sequences were analyzed in samples collected from 3552 of these healthy individuals to produce a whole-genome reference panel, 3.5KJPNv2. Allele frequency, genotype frequency, and allele count information have been made partially available to the public (https://jmorp.megabank.tohoku.ac.jp/201905).

The aims of this study were to detect genetic variants responsible for autosomal recessive bone dysplasia using 3.5KJPNv2 and to estimate the frequencies of carriers and potential patients with genetic factors among Japanese individuals.

## Materials and methods

This study was conducted after obtaining approval from the Ethics Committee of Tohoku Medical Megabank Organization of Tohoku University (authorization numbers: 2018-4-038) and written consent from all participants.

### Causative genes of bone dysplasia

We obtained information regarding bone dysplasia registered in the Surveillance Registry for Bone Dysplasia of the Japanese Orthopedic Association (https://www.joa.or.jp/)^12^. A total of 7234 cases were registered between 1990 and 2016, and were classified into 217 different diseases. The top 100 diseases with the greatest number of cases in the surveillance registry were selected, among which 30 bone dysplasias showing autosomal recessive inheritance, excluding those with autosomal dominant and X-linked inheritance, were investigated. In this study, 73 causative genes of these 30 autosomal recessive bone dysplasias were selected from among the genes registered in Nosology and Classification of Genetic Skeletal Disorders: 2015 Revision^6^ (see Table 1 for disease–gene pairs with MIM numbers). The causative genes were unknown for mesomelic dysplasia and Pyle disease among the 30 bone dysplasias. We analyzed in detail the genetic variants associated with osteogenesis imperfecta (OI), hypophosphatasia (HPP), Ellis–van Creveld syndrome (EvC), and asphyxiating thoracic dysplasia (ATD) that were reported to be prevalent in fetal bone dysplasia in obstetrics in Japan^9^. The incidence rates of these 4 diseases were obtained from a report of 448,069 patients^9^. The 95% confidence interval for the incidence rates was calculated based on the binomial distribution.

**Table 1:** The 73 genes for autosomal ressesive 30 bone dysplasias.

	Name of disorders	Gene	MIM#^a^	Phenotype MIM#^a^
1	Osteogenesis imperfecta	*BMP1*	112264	614856
		*LEPRE1*	610339	610915
		*CRTAP*	605497	610682
		*PPIB*	123841	259440
		*PLOD2*	601865	609220
		*SERPINH1*	600943	613848
		*FKBP10*	607063	610968
		*SERPINF1*	172860	613982
		*SEC24D*	607186	616294
		*WNT1*	164820	615220
		*CREB3L1*	616215	616229
		*SP7*	606633	613849
2	Hypophosphatemic rickets	*DMP1*	600980	241520
		*ENPP1*	173335	613312
		*SLC34A3*	609826	241530
3	Metaphyseal dysplasia	*POP1*	602486	617396
		*SBDS*	607444	609135
4	Mucopolysaccharidosis	*ARSB*	611542	253200
		*GALNS*	612222	253000
		*GLB1*	611458	253010
		*GNS*	607664	252940
		*GUSB*	611499	253220
		*HSGNAT*	610453	252930
		*IDUA*	252800	607014
		*NAGLU*	609701	252920
		*SGSH*	605270	252900
5	Osteopetrosis	*CA2*	611492	259730
		*CLCN7*	602727	611490
		*FERMT3*	607901	612840
		*OSTM1*	607649	259720
		*PLEKHM1*	611466	611497
		*RASGRP2*	605577	615888
		*SNX10*	614780	615085
		*TCIRG1*	604592	259700
		*TNFRSF11A*	603499	612301
		*TNFSF11*	602942	259710
6	Pyknodysostosis	*CTSK*	601105	265800
7	Spondylometaphyseal dysplasias (SMD)	*PCYT1A*	123695	608940
8	Stickler syndrome, recessive type	*COL9A1*	120210	614134
9	Spondylocostal dysostosis	*DLL3*	602768	277300
		*HES7*	608059	613686
		*LFNG*	602576	609813
		*MESP2*	605195	608681
10	Mesomelic dysplasia (Kozlowski–Reardon type)			
11	Hypophosphatasia	*ALPL*	171760	241500
12	Mucolipidosis	*GNPTAB*	607840	252500
		*GNPTG*	607838	252605
13	Ehlers–Danlos sydrome	*B4GALT7*	604327	130070
14	Chondroectodermal dysplasia (Ellis–van Creveld)	*EVC*	604831	225500
		*EVC2*	607261	225500
15	Acromesomelic dysplasia type Maroteaux (AMDM)	*NPR2*	108961	602875
16	Desbuquois dysplasia	*CANT1*	613165	251450
		*XYLT1*	608124	615777
17	Spondylo-epi-metaphyseal-dysplasias (SEMD)	*DDR2*	191311	271665
		*MATN3*	602109	608728
18	RAPADILINO syndrome Poland	*RECQL4*	603780	266280
19	Metaphyseal anadysplasia	*MMP13*	600108	250400
		*MMP9*	120361	613073
20	Antley–Bixler syndrome	*POR*	124015	201750
21	Oto-spondylo-mega-epiphyseal dysplasia (OSMED)	*COL11A2*	120290	215150
22	Diastrophic dysplasia (DTD)	*SLC26A2*	606718	222600
23	Duggve–Melchior–Clausen dysplasia (DMC)	*DYM*	607461	223800
		*RAB33B*	605950	615222
24	Dyssegmental dysplasia (Silverman–Handmaker type/Rolland–Desbuquios type)	*HSPG2*	142461	224410
25		*SLC26A2*	606718	222600
26	Progressive peudorheumatoid dysplasia (PPRD; SED with progressive arthropathy)	*WISP3*	603400	208230
27	Asphyxiating thoracic dysplasia (ATD; Jeune)	*DYNC2H1*	603297	613091
		*WDR34*	613363	615633
		*IFT80*	611177	611263
		*IFT172*	607386	615630
		*IFT140*	614620	266920
		*WDR19*	608151	614376
		*TTC21B*	612014	613819
28	Craniometaphyseal dysplasia	*GJA1*	121014	218400
29	Pyle disease			
30	Robinow syndrome	*ROR2*	602337	268310

^a^We obteined MIM# and phenotype MIM# from Online Mendelian Inheritance in Man (https://omim.org/).

### Genetic variants and annotation

We used the 3.5KJPNv2 whole-genome reference panel^10^ and the variant annotations from our previous study^13^ with subsequent updates, in which diallelic variants (after VQSR filtering) in 3.5KJPNv2 were annotated using Annovar^14^, InterVar (2.0.1)^15^, ClinVar (March 2019)^16^, and the Human Gene Mutation Database professional version (HGMD) (2019.1)^17^. InterVar is a bioinformatics tool based on the American College of Medical Genetics and Genomics (ACMG) - the Association for Molecular Pathology (AMP) variant interpretation guidelines^18^. In this study, InterVar was run using the default options and the 18 criteria for variant interpretation were used for primary interpretation.

### Classification of the genetic variants

**Fig. 1:**
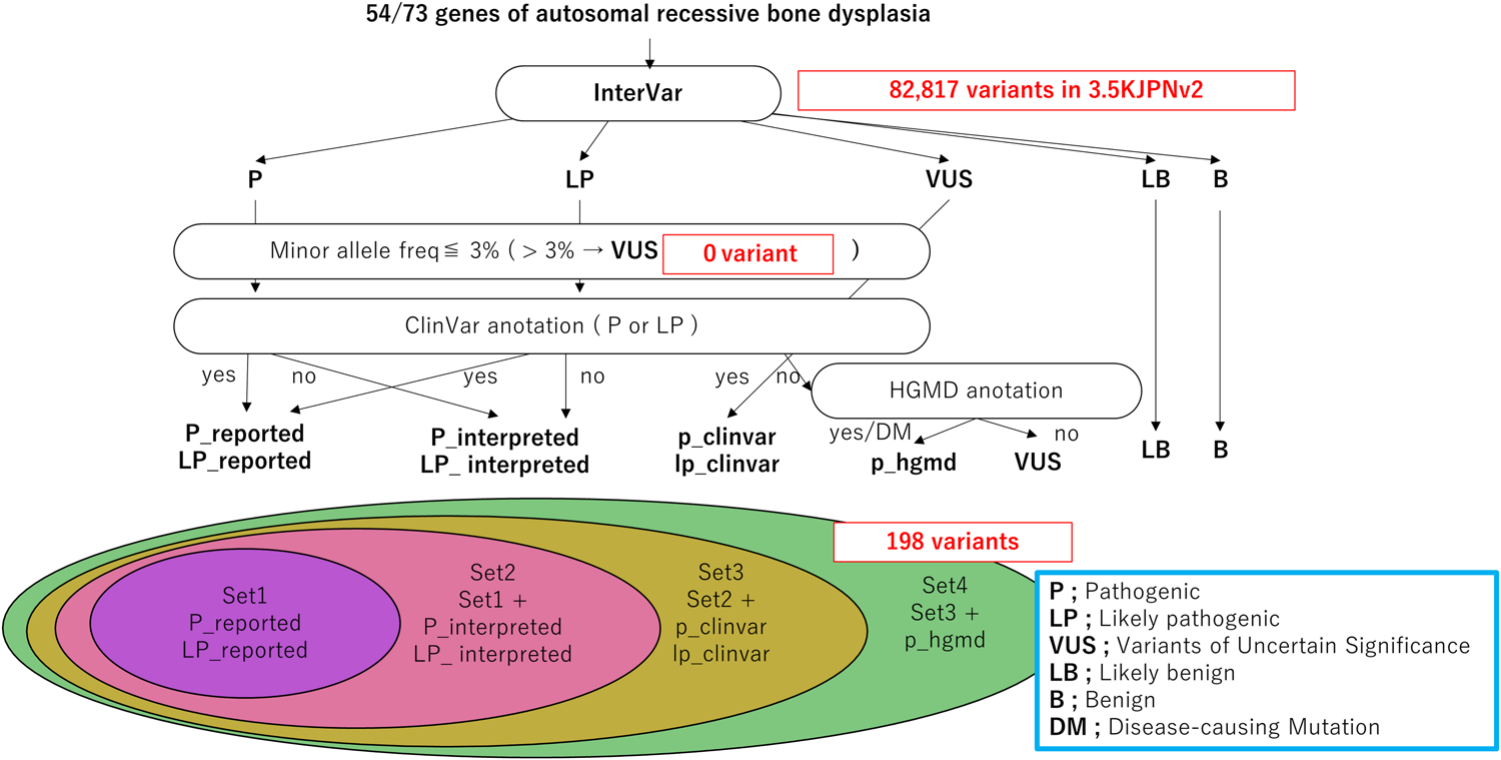
Classification of the genetic variants from the 3.5KJPNv2 panel and selection of pathogenic variants of genes for bone dysplasia. See “Materials and Methods” and the previous report^13^. The variants interpreted most conservatively as P or LP by InterVar and reported as P or LP in ClinVar were categorized into set 1. Set 2 comprises all P or LP variants, including those “reported” or “predicted (unreported).” The variants interpreted as VUS, with an MAF ≤ 0.03, or reported as P or LP in ClinVar were combined with the variants in set 2 to form set 3. Finally, set 4 comprises a combination of set 3 variants and the variants reported as disease-causing mutations (DMs) in HGMD.

In the same way as in the previous study^13^, we evaluated variants in 73 genes. First, we obtained primary interpretation by InterVar and the variants were classified into five classes: pathogenic (P), likely pathogenic (LP), variant of uncertain significance (VUS), likely benign (LB), and benign (B). Then, the pathogenic variants were examined by four different inclusion criteria (set 1–set 4)^13^ with a threshold of allele frequency (≤0.03) and correspondence to reported pathogenic variants in ClinVar and HGMD (Fig. 1).

### Estimation of the population frequency of risk alleles and expected carriers

We estimated the risk allele and carrier frequencies based on the detected pathogenic variants. First, supposing that there are *n* pathogenic variants of a gene, we calculated the sum of the risk allele frequencies at *n* sites as the estimated population frequency of pathogenic alleles of that gene (*Q*). It was assumed that the probability of having a risk allele for the disease in the haploid genome of a population was *Q* and that of not having the risk allele was *1* − *Q*. The estimated carrier frequency was then calculated as 2 × (*1* − *Q*) × *Q* based on Hardy–Weinberg equilibrium. We also calculated *Q*^2^ as the expected frequency of individuals having pathogenic variants in both chromosomes of each gene (termed the “proportion of homozygotes” in this paper) and estimated the proportion of potential patients by summing the value of *Q*^2^ for the same disease.

### Statistical analysis

A comparison of allele frequencies was performed using Excel 2016 (Microsoft, Redmond, Washington, USA). We used Fisher’s exact test to compare the allele frequencies in 3.5KJPNv2 with previously reported frequencies in the Genome Aggregation Database (gnomAD; https://gnomad.broadinstitute.org/) for Finnish (gnomAD FIN) and non-Finnish European (gnomAD NFE) individuals. The threshold for significance was *p* < 0.05.

## Results

Based on the annotation and interpretation of variants using InterVar, 82,818 genetic variants (from the 5 classifications [P, LP, VUS, LB, and B]) of 73 genes associated with 30 bone dysplasias were extracted (Fig. 1). Next, we selected potential pathogenic variants from sets 1–4 based on filtering those with a minor allele frequency (MAF) ≤ 0.03, evaluating the output of InterVar, and matching with classifications provided in ClinVar and HGMD. The total number of variants classified in sets 1–4 was 198 within 54 genes (Fig. 1). Thus, potentially pathogenic variants were detected in 54 of the 73 total genes associated with bone dysplasia in 3.5KJPNv2 but not in the remaining 19 genes (*XYLT1*, *DYM*, *RAB33B*, *DMP1*, *SLC34A3*, *MMP9*, *SBDS*, *GNS*, *CREB3L1*, *PPIB*, *SP7*, *WNT1*, *PLEKHM1*, *SNX10*, *TNFRSF11*, *TNFSF11*, *DDR2*, *HES7*, and *MESP2*) (Table 2 and Supplementary Table S1). No variant with an MAF ≤ 0.03 and classified as VUS was found (0 variant).

**Table 2:** Detection of pathogenic variants and estimated carrier frequencies of 73 genes for bone dysplasias.

Gene	Pathogenic variants in 3.5KJPNv2 (3552 individual)											
	Set 1^a^			Set 2^a^			Set 3^a^			Set 4^a^		
	No. Var	Allele Cnt	Carrier Frq.	No. Var	Allele Cnt	Carrier Frq.	No. Var	Allele Cnt	Carrier Frq.	No. Var	Allele Cnt	Carrier Frq.
Osteogenesis imperfecta												
* BMP1*	0	0	0.00000	9	9	0.00282	9	9	0.00282	9	9	0.00282
* LEPRE1*	0	0	0.00000	2	2	0.00056	2	2	0.00056	2	2	0.00056
* CRTAP*	0	0	0.00000	1	2	0.00056	1	2	0.00056	1	2	0.00056
* PPIB*	0	0	0.00000	0	0	0.00000	0	0	0.00000	0	0	0.00000
* PLOD2*	0	0	0.00000	2	2	0.00056	2	2	0.00056	2	2	0.00056
* SERPINH1*	0	0	0.00000	8	22	0.00618	8	22	0.00618	8	22	0.00618
* FKBP10*	0	0	0.00000	1	1	0.00028	1	1	0.00028	1	1	0.00028
* SERPINF1*	0	0	0.00000	1	1	0.00141	1	1	0.00141	2	91	0.02721
* SEC24D*	0	0	0.00000	1	1	0.00028	1	1	0.00028	1	1	0.00028
* WNT1*	0	0	0.00000	0	0	0.00000	0	0	0.00000	0	0	0.00000
* CREB3L1*	0	0	0.00000	0	0	0.00000	0	0	0.00000	0	0	0.00000
* SP7*	0	0	0.00000	0	0	0.00000	0	0	0.00000	0	0	0.00000
Hypophosphatemic rickets												
* DMP1*	0	0	0.00000	0	0	0.00000	0	0	0.00000	0	0	0.00000
* ENPP1*	0	0	0.00000	0	0	0.00000	0	0	0.00000	2	5	0.00141
* SLC34A3*	0	0	0.00000	0	0	0.00000	0	0	0.00000	0	0	0.00000
Metaphyseal dysplasia												
* POP1*	0	0	0.00000	2	3	0.00085	2	3	0.00085	2	3	0.00085
* SBDS*	0	0	0.00000	0	0	0.00000	0	0	0.00000	0	0	0.00000
*Mucopolysaccharidosis*												
* ARSB*	1	1	0.00028	1	1	0.00028	1	1	0.00028	1	1	0.00028
* GALNS*	1	2	0.00056	4	4	0.00113	4	4	0.00113	5	5	0.00141
* GLB1*	3	3	0.00085	5	5	0.00141	5	5	0.00141	6	6	0.00169
* GNS*	0	0	0.00000	0	0	0.00000	0	0	0.00000	0	0	0.00000
* GUSB*	0	0	0.00000	1	1	0.00028	1	1	0.00028	2	2	0.00056
* HSGNAT*	0	0	0.00000	1	1	0.00028	1	1	0.00028	2	2	0.00056
* IDUA*	1	1	0.00028	3	3	0.00085	4	4	0.00113	5	5	0.00141
* NAGLU*	1	1	0.00028	2	2	0.00056	2	2	0.00056	3	3	0.00085
* SGSH*	0	0	0.00000	1	1	0.00028	1	1	0.00028	2	2	0.00056
Osteopetrosis												
* CA2*	1	1	0.00028	1	1	0.00028	1	1	0.00028	1	1	0.00028
* CLCN7*	0	0	0.00000	9	13	0.00366	9	13	0.00366	9	13	0.00366
* FERMT3*	0	0	0.00000	3	4	0.00113	3	4	0.00113	3	4	0.00113
* OSTM1*	0	0	0.00000	1	1	0.00028	1	1	0.00028	1	1	0.00028
* PLEKHM1*	0	0	0.00000	0	0	0.00000	0	0	0.00000	0	0	0.00000
* RASGRP2*	0	0	0.00000	2	2	0.00056	2	2	0.00056	2	2	0.00056
* SNX10*	0	0	0.00000	0	0	0.00000	0	0	0.00000	0	0	0.00000
* TCIRG1*	0	0	0.00000	0	0	0.00000	1	1	0.00028	2	2	0.00056
* TNFRSF11A*	0	0	0.00000	0	0	0.00000	0	0	0.00000	0	0	0.00000
* TNFSF11*	0	0	0.00000	0	0	0.00000	0	0	0.00000	0	0	0.00000
Pyknodysostosis												
* CTSK*	1	2	0.00056	2	3	0.00085	2	3	0.00085	2	3	0.00085
Spondylometaphyseal dysplasias (SMD)												
* PCYT1A*	2	2	0.00056	2	2	0.00056	2	2	0.00056	2	2	0.00056
Stickler syndrome, recessive type												
* COL9A1*	1	1	0.00028	3	3	0.00113	3	3	0.00113	3	3	0.00113
Spondylocostal dysostosis												
* DLL3*	1	1	0.00028	2	2	0.00056	2	2	0.00056	2	2	0.00056
* HES7*	0	0	0.00000	0	0	0.00000	0	0	0.00000	0	0	0.00000
* LFNG*	0	0	0.00000	0	0	0.00000	0	0	0.00000	1	1	0.00028
* MESP2*	0	0	0.00000	0	0	0.00000	0	0	0.00000	0	0	0.00000
Hypophosphatasia												
* ALPL*	2	18	0.00506	5	22	0.00618	6	34	0.00953	13	156	0.04404
Mucolipidosis												
* GNPTAB*	5	8	0.00225	6	9	0.00253	6	9	0.00253	6	9	0.00253
* GNPTG*	0	0	0.00000	1	1	0.00028	1	1	0.00028	1	1	0.00028
Ehlers–Danlos sydrome												
* B4GALT7*	0	0	0.00000	1	1	0.00028	1	1	0.00028	1	1	0.00028
Chondroectodermal dysplasia (Ellis–van Creveld)												
* EVC*	0	0	0.00000	1	1	0.00028	1	1	0.00028	3	10	0.00281
* EVC2*	2	3	0.00085	7	8	0.00226	7	8	0.00226	8	9	0.00254
Acromesomelic dysplasia type Maroteaux (AMDM)												
* NPR2*	0	0	0.00000	1	1	0.00028	2	5	0.00141	2	5	0.00141
Desbuquois dysplasia												
* CANT1*	0	0	0.00000	2	3	0.00085	3	6	0.00169	3	6	0.00169
* XYLT1*	0	0	0.00000	0	0	0.00000	0	0	0.00000	0	0	0.00000
Spondylo-epi-metaphyseal-dysplasias (SEMD)												
* DDR2*	0	0	0.00000	0	0	0.00000	0	0	0.00000	0	0	0.00000
* MATN3*	0	0	0.00000	0	0	0.00000	0	0	0.00000	2	17	0.00478
RAPADILINO syndrome Poland												
* RECQL4*	2	2	0.00056	6	9	0.00253	7	10	0.00281	7	10	0.00281
Metaphyseal anadysplasia												
* MMP13*	0	0	0.00000	1	3	0.00084	1	3	0.00084	1	3	0.00084
* MMP9*	0	0	0.00000	0	0	0.00000	0	0	0.00000	0	0	0.00000
Antley–Bixler syndrome												
* POR*	3	15	0.00422	5	17	0.00478	5	17	0.00478	8	41	0.01148
Oto-spondylo-mega-epiphyseal dysplasia (OSMED)												
* COL11A2*	0	0	0.00000	4	4	0.00113	4	4	0.00113	5	6	0.00169
Diastrophic dysplasia (DTD)/ Atelosteogenesis type2 (AO2)												
* SLC26A2*	0	0	0.00000	0	0	0.00000	1	2	0.00056	2	14	0.00393
Duggve–Melchior–Clausen dysplasia (DMC)												
* DYM*	0	0	0.00000	0	0	0.00000	0	0	0.00000	0	0	0.00000
* RAB33B*	0	0	0.00000	0	0	0.00000	0	0	0.00000	0	0	0.00000
Dyssegmental dysplasia (Silverman–Handmaker type/Rolland–Desbuquios type)												
* HSPG2*	0	0	0.00000	6	7	0.00197	6	7	0.00197	9	16	0.00450
Progressive peudorheumatoid dysplasia (PPRD; SED with progressive arthropathy)												
* WISP3*	0	0	0.00000	1	1	0.00028	1	1	0.00028	1	1	0.00028
Asphyxiating thoracic dysplasia (ATD; Jeune)												
* DYNC2H1*	2	4	0.00113	6	8	0.00225	6	8	0.00225	7	12	0.00337
* WDR34*	0	0	0.00000	1	1	0.00028	1	1	0.00028	1	1	0.00028
* IFT80*	0	0	0.00000	3	14	0.00393	3	14	0.00393	3	14	0.00393
* IFT172*	1	1	0.00028	5	5	0.00141	5	5	0.00141	5	5	0.00141
* IFT140*	0	0	0.00000	3	3	0.00085	3	3	0.00085	5	5	0.00141
* WDR19*	1	2	0.00056	4	6	0.00169	4	6	0.00169	4	6	0.00169
* TTC21B*	0	0	0.00000	4	5	0.00141	4	5	0.00141	6	7	0.00197
Craniometaphyseal dysplasia												
* GJA1*	0	0	0.00000	1	1	0.00028	1	1	0.00028	1	1	0.00028
Robinow syndrome												
* ROR2*	0	0	0.00000	1	1	0.00028	1	1	0.00028	2	2	0.00056

^a^See “Materials and Methods” and Fig. 1.

After the detection of pathogenic variants, we examined the status of individuals using individual genotype data from 3.5KJPNv2. Of note, we checked whether there was any compound heterozygote or homozygote for a single gene. There were two exceptional individuals with multiple pathogenic variants in single genes and we manually inspected the cases of these two individuals. One individual had seven predicted pathogenic variants of the *RECQL4* gene. The seven variants are indels located in a short region (67 bp, Chr7:145737572–145737639) and all were singletons. Through the inspection of the status of mapped reads in BAM files (binary format of sequence alignment map)^10^ using the Integrative Genomic Viewer, we found that variant calls by HaplotypeCaller were not successful for this local region in the individual. Thus, we did not use these variants in further analysis of the estimation of frequency. Another individual had 2 predicted pathogenic variants in the *BMP1* gene. These two variants are 1 bp (A) deletions at very close (3 bp) genomic sites (chr8:22034590 and chr8:22034593). They were singleton variants and were located on the same chromosome of the individual. Thus, these two variants could be a single variant. After corrections based on these two exceptional cases, the proportions (and number) of individuals having at least one pathogenic variant were 1.89% (67), 6.28% (222), 6.95% (247), and 15.0% (534) for sets 1–4, respectively. The number of individuals having 2 pathogenic variants was 1, 7, 7, and 25 for sets 1–4, respectively, and 2 individuals in set 4 had 3 pathogenic variants.

The carrier frequencies were estimated based on Hardy–Weinberg equilibrium using the allele frequencies of the variants associated with OI, EvC, ATD, and HPP (Tables 2 and 3). We calculated the expected frequency of individuals with homozygous variants from the estimated carrier frequency, estimated the proportions of potential patients, and compared them with the reported frequency in Japan (Table 4).

**Table 3:** Possible pathogenic variants in the genes of OI, HPP, EvC, and ATD in 3.5KJPNv2.

Variation	Functional category	Genomic location	dbSNP	3.5KJPN genotype count			3.5KJPN allele freq	InterVar	gnomAD ALL^a^	gnomAD FIN^a^	gnomAD NFE	Mutation database		Classification	Set
		Position (GRCh37/hg19)		RefRef	Het	AltAlt						ClinVar	HGMD		
Osteogenesis imperfecta (OI)															
* BMP1*															
c.G1639T:p.Glu547^a^	Stopgain	chr8:22052432		3551	1	0	0.00014	P						P_interpreted	2
c.2826 + 2 T > C	Splicing	chr8:22067210		3551	1	0	0.00014	P						P_interpreted	2
c.G626T:p.Gly209Val	Nonsynonymous SNV	chr8:22034548		3551	1	0	0.00014	LP						LP_interpreted	2
c.668delA:p.His223fs	Frameshift deletion	chr8:22034590		3551	1	0	0.00014	LP						LP_interpreted	2
c.671delA:p.Glu224fs	Frameshift deletion	chr8:22034593		3551	1	0	0.00014	LP						LP_interpreted	2
c.C831A:p.Phe277Leu	Nonsynonymous SNV	chr8:22035465		3551	1	0	0.00014	LP						LP_interpreted	2
c.G850T:p.Asp284Tyr	Nonsynonymous SNV	chr8:22037231		3551	1	0	0.00014	LP						LP_interpreted	2
c.A1640G:p.Glu547GLy	Nonsynonymous SNV	chr8:22052975	rs764357220	3551	1	0	0.00014	LP						LP_interpreted	2
c.G1757A:p.Arg586His	Nonsynonymous SNV	chr8:22053092	rs755936351	3551	1	0	0.00014	LP	3.24E-05	0.0003	0			LP_interpreted	2
c.Gly2137A:p.Gly713Ser	Nonsynonymous SNV	chr8:22059345	rs148687489	3551	1	0	0.00014	LP	0.0002	0	0			LP_interpreted	2
* LEPRE1*															
c.C1726T:p.Gln576^a^	Stopgain	chr1:43213983		3551	1	0	0.00014	P						P_interpreted	2
c.G1840Ala:p.Ala614Thr	Nonsynonymous SNV	chr1:43213468	rs868224632	3551	1	0	0.00014	P						LP_interpreted	2
* CRTAP*															
c.621 + 1 G > A	Splicing	chr3:33161986	rs775720622	3550	2	0	0.00028	P						P_interpreted	2
*PPIB*															
PLOD2															
c.C1483T:p.Arg495^a^	Stopgain	chr3:145796920		3551	1	0	0.00014	P						P_interpreted	2
c.C160T:p.Arg54^a^	Stopgain	chr3:145841966	rs780902890	3551	1	0	0.00014	P						P_interpreted	2
* SERPINH1*															
c.C230T:p.Ser77Leu	Nonsynonymous SNV	chr11:75277624		3543	9	0	0.00127	LP						LP_interpreted	2
c.G655A:p.Val219Met	Nonsynonymous SNV	chr11:75279808		3550	2	0	0.00028	LP						LP_interpreted	2
c.C680Thr:p.Thr227Ile	Nonsynonymous SNV	chr11:75279833		3551	1	0	0.00014	LP						LP_interpreted	2
c.A731T:p.Asn244Ile	Nonsynonymous SNV	chr11:75279993		3550	2	0	0.00028	LP						LP_interpreted	2
c.G766A:p.Val256Met	Nonsynonymous SNV	chr11:75280028	rs749664592	3551	1	0	0.00014	LP						LP_interpreted	2
c.G1016T:p.Arg339Leu	Nonsynonymous SNV	chr11:75282887	rs535510332	3551	1	0	0.00014	LP						LP_interpreted	2
c.C1046T:p.Ala349Val	Nonsynonymous SNV	chr11:75282917		3547	5	0	0.00070	LP						LP_interpreted	2
c.G1060Ala:p.Ala354Thr	Nonsynonymous SNV	chr11:75282931	rs369550626	3551	1	0	0.00014	LP	3.23E-05	0	0			LP_interpreted	2
* FKBP10*															
c.G1723C:p.Glu575Gln	Nonsynonymous SNV	chr17:39978634		3551	1	0	0.00014	LP						LP_interpreted	2
* SERPINF1*															
c.284-2 A > G	Splicing	chr17:1674321	rs113947687	3547	5	0	0.00070	P						P_interpreted	2
c.C167Gly:p.Ala56Gly	Nonsynonymous SNV	chr17:1673228	rs76119062	3465	86	1	0.01239	VUS	0.0004	0.0017	6.68E-05		DM	p_hgmd	4
* SEC24D*															
c.C904T:p.Gln302^a^	Stopgain	chr4:119727007	rs770892912	3551	1	0	0.00014	P						P_interpreted	2
* WNT1*															
* CREB3L1*															
* SP7*															
Hypophosphatasia (HPP)															
* ALPL*															
c.G407A:p.Arg136His	Nonsynonymous SNV	chr1:21889712	rs121918011	3550	2	0	0.00028	LP	3.23E-05	0	0	P	DM	LP_reported	1
c.T979C:p.Phe327Leu	Nonsynonymous SNV	chr1:21900274	rs121918010	3536	16	0	0.00225	LP	9.69E-05	0	0	P	DM	LP_reported	1
c.C98Gly:p.Ala33Gly	Nonsynonymous SNV	chr1:21887155	rs121918005	3551	1	0	0.00014	LP						LP_interpreted	2
c.C782A:p.Pro261Gln	Nonsynonymous SNV	chr1:21894730	rs765149569	3550	2	0	0.00028	LP						LP_interpreted	2
c.A1183G:p.Ile395Val	Nonsynonymous SNV	chr1:21902411	rs772682471	3551	1	0	0.00014	LP					DM	LP_interpreted	2
c.1559delT:p.Leu520fs	Frameshift deletion	chr1:21904125	rs387906525	3540	12	0	0.00169	VUS				P	DM	p_clinvar	3
c.A184G:p.Met62Val	Nonsynonymous SNV	chr1:21887592		3551	1	0	0.00014	VUS					DM	p_hgmd	4
c.G529Ala:p.Ala177Thr	Nonsynonymous SNV	chr1:21890590	rs199669988	3434	116	2	0.01689	VUS	6.47E-05	0	0	VUS	DM	p_hgmd	4
c.A572G:p.Glu191Gly	Nonsynonymous SNV	chr1:21890633		3551	1	0	0.00014	VUS					DM	p_hgmd	4
c.A1022G:p.His341Arg	Nonsynonymous SNV	chr1:21902250		3551	1	0	0.00014	VUS	3.23E-05	0.0003	0		DM	p_hgmd	4
c.Gly1258A:p.Gly420Ser	Nonsynonymous SNV	chr1:21903083		3551	1	0	0.00014	VUS					DM	p_hgmd	4
c.Gly1276A:p.Gly426Ser	Nonsynonymous SNV	chr1:21903101	rs770548228	3551	1	0	0.00014	VUS					DM	p_hgmd	4
c.A1307G:p.Tyr436Cys	Nonsynonymous SNV	chr1:21903132		3551	1	0	0.00014	VUS					DM	p_hgmd	4
Ellis–van Creveld syndrome (EvC)															
* EVC*															
c.1887-1 G > C	Splicing	chr4:5798748		3551	1	0	0.00014	P						P_interpreted	2
c.C884G:p.Thr295Ser	Nonsynonymous SNV	chr4:5747013	rs754532508	3550	2	0	0.00028	VUS	3.24E-05	0	0	VUS	DM	p_hgmd	4
c.C982T:p.Leu328Phe	Nonsynonymous SNV	chr4:5749917	rs199916502	3545	7	0	0.00099	VUS	0.0004	0	6.66E-05	Conflicting interpretations of pathogenicity	DM	p_hgmd	4
* EVC2*															
c.G2484A:p.Trp828^a^	Stopgain	chr4:5624281	rs770918273	3551	1	0	0.00014	P	3.24E-05	0	0	LP	DM	P_reported	1
c.C1195T:p.Arg399^a^	Stopgain	chr4:5642516	rs137852924	3550	2	0	0.00028	P	6.46E-05	0	0	P	DM	P_reported	1
c.1230 + 1 G > C	Splicing	chr4:5642240		3551	1	0	0.00014	P					DM	P_interpreted	2
c.906-2 A > C	Splicing	chr4:5642567		3551	1	0	0.00014	P					DM	P_interpreted	2
c.2536delG:p.Glu846fs	Frameshift deletion	chr4:5620375		3551	1	0	0.00014	LP					DM	LP_interpreted	2
c.C2092T:p.Arg698^a^	Stopgain	chr4:5624673	rs781623802	3551	1	0	0.00014	LP					DM	LP_interpreted	2
c.1082dupA:p.Asn361fs	Frameshift insertion	chr4:5664896		3551	1	0	0.00014	LP					DM	LP_interpreted	2
c.C2848T:p.Arg950Trp	Nonsynonymous SNV	chr4:5586559	rs137852928	3551	1	0	0.00014	VUS				VUS	DM	p_hgmd	4
Asphyxiation thoracic dystrophy (ATD)															
* DYNC2H1*															
c.5681_5682del:p.Glu1894fs	Frameshift deletion	chr11:103046970-103046971	rs767846762	3549	3	0	0.00042	P				P	DM	P_reported	1
c.C10045T:p.Arg3349^a^	Stopgain	chr4:103116085	rs751891969	3551	1	0	0.00014	P				P		P_reported	1
c.11277 + 1 G > A	splicing	chr4:103173983		3551	1	0	0.00014	P						P_interpreted	2
c.5518_5519insTA:p.Val1840fs	Frameshift insertion	chr4:103043994		3551	1	0	0.00014	LP						LP_interpreted	2
c.8062delA:p.Lys2688fs	Frameshift deletion	chr4:103070179		3551	1	0	0.00014	LP						LP_interpreted	2
c.9977delG:p.Arg3326fs	Frameshift deletion	chr4:103116017		3551	1	0	0.00014	LP						LP_interpreted	2
c.Cys9010T:p.Arg3004Cys	Nonsynonymous SNV	chr4:103091415		3548	4	0	0.00056	VUS	3.23E-05	0.0003	0		DM	p_hgmd	4
* WDR34*															
c.1372 + 2 T > C	Splicing	chr9:131396503	rs758936528	3551	1	0	0.00014	P						P_interpreted	2
* IFT80*															
c.C401G:p.Ser134^a^	Stopgain	chr3:160093638		3545	7	0	0.00099	P						P_interpreted	2
c.371-1 G > C	Splicing	chr3:160093669	rs769745055	3546	6	0	0.00085	P						P_interpreted	2
c.40-1 T > C	Splicing	chr3:160099511		3551	1	0	0.00014	P						P_interpreted	2
* IFT172*															
c.C811T:p.Arg271^a^	Stopgain	chr2:27702991		3551	1	0	0.00014	P				P		P_reported	1
c.3229-1 G > C	Splicing	chr2:27679521		3551	1	0	0.00014	P						P_interpreted	2
c.2116-1 G > A	Splicing	chr2:27684704		3551	1	0	0.00014	P						P_interpreted	2
c.571-1 G > A	Splicing	chr2:27704128	rs775935517	3551	1	0	0.00014	P						P_interpreted	2
c.1989delC:p.Thr663fs	Frameshift deletion	chr2:27685997		3551	1	0	0.00014	LP						LP_interpreted	2
* IFT140*															
c.C2992T:p.Gln998^a^	Stopgain	chr16:1574790		3551	1	0	0.00014	P						P_interpreted	2
c.C3214T:p.Arg1072^a^	Stopgain	chr16:1573885		3551	1	0	0.00014	LP						LP_interpreted	2
c.2767_2768del:p.Tyr923fs	Frameshift deletion	chr16:1575886-1575889	rs769075694	3551	1	0	0.00014	LP						LP_interpreted	2
c.G4182C:p.Thr1394Thr	Synonymous SNV	chr16:1568217		3551	1	0	0.00014	VUS					DM	p_hgmd	4
c.C489T:p.Gly163Gly	Synonymous SNV	chr16:1642470	rs776597097	3551	1	0	0.00014	VUS					DM	p_hgmd	4
* WDR19*															
c.634dupT:p.Leu211fs	Frameshift insertion	chr4:39206803	rs587777348	3550	2	0	0.00028	P				P	DM	P_reported	1
c.2165 + 1 G > T	Splicing	chr4:39246173		3551	1	0	0.00014	P					DM	P_interpreted	2
c.2782-2 A > G	Splicing	chr4:39269613	rs753291151	3550	2	0	0.00028	P						P_interpreted	2
c.Gly2365C:p.Gly789Arg	Nonsynonymous SNV	chr4:39241898		3551	1	0	0.00014	LP						LP_interpreted	2
* TTC21B*															
c.G1111T:p.Glu371^a^	Stopgain	chr2:166786234		3551	1	0	0.00014	P						P_interpreted	2
c.430-2 A > C	Splicing	chr2:166799853		3551	1	0	0.00014	P						P_interpreted	2
c.3845_3846del:p.Tyr1282fs	Frameshift deletion	chr2:166732702-166732703		3551	1	0	0.00014	LP						LP_interpreted	2
c.3224_3225insGAAACTGT:p.Val1075fs	Frameshift insertion	chr2:166747027		3550	2	0	0.00028	LP						LP_interpreted	2
c.Cys2599T:p.Arg867Cys	Nonsynonymous SNV	chr2:166758390	rs746700857	3551	1	0	0.00014	VUS	3.24E-05	0	0		DM	p_hgmd	4
c.A1697G:p.His566Arg	Nonsynonymous SNV	chr2:166773969	rs146320075	3551	1	0	0.00014	VUS	0.0014	0.0023	0.0021	VUS	DM	p_hgmd	4

*B* benign, *DM* disease-causing mutation, *FIN* Finnish, *gnomAD* gnomAD the Genome Aggregation Database (https://gnomad.broadinstitute.org/), *LB* likely benign, *LP* likely pathogenic, *NFE* non-Finnish European, *P* pathogenic, *VUS* variant of significant.

^a^See “Materials and Methods” and Fig. 1.

**Table 4:** Estimated proportion of homozygotes of pathogenic variants for four bone dysplasias.

Four clinically important bone dysplasia	Gene	Estimated proportion of homozygotes^a^				Reported incidence rates		
		Set 1^b^	Set 2^b^	Set 3^b^	Set 4^b^	Incidence rates^c^	Lower limit^d^	Upper limit^d^
OI	*BMP1*, *LEPRE1*, *CRTAP*, *PLOD2*, *SERPINH1*, *FKBP10*, *SERPINF1*, *SEC24D*	0	4.8.E − 05	4.8.E − 05	2.2.E − 04	4.5.E − 05	2.5.E − 05	6.4.E − 05
HPP	*ALPL*	2.6.E − 05	6.4.E − 05	1.5.E − 04	6.6.E − 04	1.6.E − 05	4.0.E − 06	2.7.E − 05
ATD	*DYNC2H1*, *WDR34*, *IFT80*, *IFT172*, *IFT140*, *WDR19*, *TTC21B*	1.7.E − 06	2.8.E − 05	2.8.E − 05	3.8.E − 05	1.6.E − 05	4.0.E − 06	2.7.E − 05
EVC	*EVC*, *EVC2*	7.2.E − 07	5.2.E − 06	5.2.E − 06	1.4.E − 05	2.2.E − 06	−2.1.E − 06	6.6.E − 06

^a^We calculated the total frequency of homozygotes, by summing the expected homozygotes of pathogenic variants (*Q*^2^) for each causative gene.

^b^See “MaterialS and MethodS,” and Fig. 1 for variant classification and selection.

^c^Incidence rates of the four diseases were from a previous report^10^ in 448,069 patients.

^d^The 95% confidence interval was calculated based on the binomial distribution.

### Osteogenesis imperfecta

#### *BMP1* (MIM# 614856)

A stopgain variant, p.Glu547*, and a splicing variant, c.2826 + 2 T > C, were automatically classified as P by InterVar. A frameshift variant, p.His223fs (p.Glu224fs), was automatically classified as LP by InterVar. The remaining six nonsynonymous SNVs were automatically classified as LP by InterVar. One individual was heterozygous for all ten variants and the allele frequency was 0.00014 for each variant (Table 3). None had been reported in ClinVar or HGMD and, thus, these variants were included in our set 2 (Fig. 1). The carrier frequency of the *BMP1* variants was estimated to be 0.00253 in set 2 (Table 2).

#### *CRTAP* (MIM# 610682)

A splicing variant, c.621 + 1 G > A, was interpreted as P by InterVar and included in set 2, although it is not reported in ClinVar or HGMD (Table 3). The allele frequency was 0.00028 and the carrier frequency of *CRTAP* was estimated to be 0.00056 in set 2 (Table 2).

#### *LEPRE1* (*P3H1*) (MIM# 610915)

A stopgain variant, p.Gln576*, and a nonsynonymous single nucleotide variant (SNV), p.Ala614Thr, were detected in one heterozygous individual, each with an allele frequency of 0.00014, and these variants were interpreted as P by InterVar and thus included in set 2 (Table 3). A nonsynonymous SNV, p.Ala614Thr, was interpreted as LP by InterVar and thus included in set 2 (Table 3). The estimated carrier frequency of *LEPRE1* was 0.00056 in set 2 (Table 2).

#### *FKBP10* (MIM # 610968)

A nonsynonymous SNV, p.Glu575Gln, of *FKBP10* was interpreted as LP by InterVar and included in our set 2, although it is not reported in ClinVar or HGMD (Table 3). The allele frequency was 0.00014 and the carrier frequency was 0.00028 in set 2 (Table 2).

#### *SERPINH1* (MIM# 613848)

All eight variants were nonsynonymous variants and were interpreted as LP by InterVar, and thus included in set 2; none are reported in ClinVar or HGMD (Table 3). Of the eight nonsynonymous variants, all were heterozygous, with p.Thr227Ile, p.Val256Met, p.Arg339Leu, and p.Ala354Thr identified in one individual, each with an allele frequency of 0.00014; p.Ser77Leu was identified in nine individuals, with an allele frequency of 0.00127; p.Val219Met and p.Asn244Ile was identified in two individuals, each with an allele frequency of 0.00028; and p.Ala349Val was identified in five individuals, with an allele frequency of 0.0007. The carrier frequency was 0.00618 in set 2 (Table 2).

#### *PLOD2* (MIM# 609220)

Two stopgain variants, p.Arg495* and p.Arg54*, were not reported in ClinVar or HGMD and were interpreted as P by InterVar and included in our set 2 (Table 3). These variants were found in one heterozygous individual. The allele frequency was 0.00014 and the carrier frequency was 0.00056 in set 2 (Table 2).

#### *SERPINF1* (MIM# 613848)

A splicing variant, c.284-2 A > G, was detected in five heterozygous individuals with an allele frequency of 0.0007. This variant was interpreted as P by InterVar. However, it was not reported in ClinVar or HGMD (Table 3). A nonsynonymous SNV, p.Ala56Gly, was heterozygous in 86 individuals with an allele frequency of 0.01239, which is much higher than that reported in gnomAD in European subjects (*p* < 0.001, Table 3). This variant was reported in HGMD, so it was included in our set 4. The carrier frequencies were 0.00141 in set 2 and 0.02584 in set 4 (Table 2).

#### *SEC24D* (MIM# 616295)

A stopgain variant, p.Gln302*, was identified in one heterozygous individual in 3.5KJPNv2 and was interpreted as P by InterVar, and thus included in our set 2 (Table 3). The allele frequency was 0.00014 and the carrier frequency was 0.00028 in set 2 (Table 2).

Of the 12 causative genes registered in the international classification of autosomal recessive OI, 4 genes (*PPIB*, *WNT1*, *CREB3L1*, and *SP7*) were not recognized as pathogenic variants in this study (Tables 2 and 3). Except for p.Ala56Gly in *SERPINF1*, none of the pathogenic variants of the eight genes detected in this study was registered in ClinVar or HGMD. They were pathogenic variants detected for the first time in association with OI in this study. Most of the 12 OI-associated variant genes were included in set 2 and p.Ala56Gly was included in set 4, with a total carrier frequency of 0.01238 (set 2) and 0.03681 (set 4). The expected proportions of potential patients calculated by allele frequencies were 1/20,967 (set 2) and 1/4474 (set 4) (Table 4).

### Hypophosphatasia

Thirteen pathogenic variants of *ALPL* (MIM# 241500) were detected in 3.5KJPNv2. Two nonsynonymous SNVs, p.Arg136His and p.Phe327Leu, were classified as LP by InterVar and reported as P in ClinVar and DM in HGMD. Thus, they were included in our set 1 (Table 3 and Fig. 1). The allele frequency of p.Arg136His was 0.00028 and this variant was identified in 2 heterozygous individuals, whereas the allele frequency of p.Phe327Leu was 0.00225 and this variant was identified in 16 individuals. Three nonsynonymous SNVs, p.Ala33Gly, p.Pro261Gln, and p.Ile395Val, were included in our set 2, because they were classified as LP by InterVar but not reported in ClinVar or HGMD (Table 3). Two variants, p.Ala33Gly and p.Ile395Val, were identified in one heterozygous individual and p.Pro261Gln was detected in two individuals. A frameshift variant, c.1559delT, was reported as P in ClinVar and DM in HGMD but classified as VUS by InterVar. This variant was classified in our set 3 and it was identified in 12 heterozygous individuals. Seven nonsynonymous SNVs, p.Met62Val, p.Ala177Thr, p.Glu191Gly, p.His341Arg, p.Gly420Ser, p.Gly426Ser, and p.Tyr436Cys, were classified as VUS by InterVar and DM in HGMD. These variants were classified in our set 4. Six variants, p.Arg136His, p.Glu191Gly, p.Phe327Leu, p.His341Arg, p.Gly426Ser, and c.1559delT, have been reported in the Japanese population^19,20^. Among these variants, p.Phe327Leu and c.1559delT were identified in 16 and 12 heterozygous individuals, respectively, and p.Ala177Thr was identified in 116 heterozygous individuals, with an allele frequency of 0.01689, which is much higher than that in gnomAD in European subjects (*p* < 0.001, Table 3). The carrier frequencies estimated from the sum of the allele frequencies were 0.00506 (set 1), 0.00618 (set 2), 0.00953 (set 3), and 0.04404 (set 4) (Table 2). The expected proportions of potential patients were 1/39,132 (set 1), 1/15,697 (set 2), 1/6473 (set 3), and 1/1511 (set 4) (Table 4).

### Ellis–van Creveld syndrome

#### *EVC* (MIM# 225500)

One individual was heterozygous for the splicing variant c.1887-1 G > C, which was not reported in ClinVar or HGMD. This variant was interpreted as P by InterVar; thus, we included it in set 2 (Table 3 and Fig. 1). The allele frequency was 0.000141. Although two nonsynonymous variants, p.Thr295Ser and p.Leu328Phe, were considered DM in HGMD, they were interpreted as VUS by InterVar; thus, we included them in set 4. Both variants were identified in genetic testing of patients with fetal limb shortening^21^. We identified 2 and 7 heterozygous individuals for p.Thr295Ser and p.Leu328Phe, respectively. The allele frequencies of p.Thr295Ser and p.Leu328Phe were 0.00028 and 0.000985, respectively, which were higher than those in gnomAD in European subjects (*p* < 0.05). The carrier frequencies were 0.00028 in set 2 and 0.00281 in set 4 (Table 2).

#### *EVC2* (MIM# 225500)

A stopgain variant, p.Arg399*, was heterozygous in two individuals. This variant was reported as P both by InterVar and in ClinVar but as DM in HGMD; we included it in set 1 (Table 3). This variant was detected in stillborn children with a ventricular septal defect and limb shortening with polydactyly^22^. A stopgain variant, p.Trp828*, was detected as heterozygous in one individual. Because it was reported as P by InterVar, as DM in HGMD and as LP in ClinVar, we included it in set 1. This was reported in genetic testing of a boy with distal limb shortening and polydactyly^23^. Two splicing variants, c.1230 + 1 G > C and c.906-2 A > C, were interpreted as P by InterVar and categorized in set 2, although they were not reported in ClinVar or HGMD. They were detected as heterozygous in one individual each and the allele frequencies of both were 0.00014. Two frameshift variants, p.Glu846fs and p.Asn361fs, were interpreted as LP by InterVar and included in set 2, although neither is reported in ClinVar or HGMD. Each was heterozygous in one individual, with an allele frequency of 0.00014. A stop codon, p.Arg698*, was included in set 2 according to InterVar and identified as heterozygous in one individual. A nonsynonymous variant, p.Arg950Trp, was reported as DM in HGMD but as VUS in ClinVar and InterVar by default. Therefore, p.Arg950Trp was included in set 4. This variant was identified in one heterozygous individual, with an allele frequency of 0.00014. The carrier frequencies of *EVC2* were estimated to be 0.00085 in set 1, 0.00225 in set 2, and 0.00253 in set 4 (Table 2).

The sums of the estimated carrier frequencies of *EVC* and *EVC2* were 0.00085 (set 1), 0.00254 (set 2), and 0.00535 (set 4). The proportions of potential patients calculated by allele frequencies were 1/1,398,385 (set 1), 1/193,890 (set 2), and 1/69,771 (set 4) (Table 4).

### Asphyxiating thoracic dysplasia

#### *DYNC2H1* (MIM# 613091)

A frameshift variant, p.Glu1894fs, and a stopgain variant, p.Arg3349* was classified in set 1 because they were interpreted as P by InterVar and reported as P in ClinVar (Table 3 and Fig. 1). Three individuals were identified to be heterozygous for p.Glu1894fs and 1 individual was identified to be heterozygous for p.Arg3349*. A splicing variant, c.11277 + 1 G > A, which was interpreted as P by InterVar, and three frameshift variants, p.Val1840fs, p.Lys2688fs, and p.Arg3326fs, which were interpreted as LP by InterVar, were classified in set 2. However, they were not reported in ClinVar or HGMD. Each was identified in one heterozygous individual. A nonsynonymous variant, p.Arg3004Cys, was identified in 4 heterozygous individuals. This variant was reported as DM in HGMD and was identified in a Japanese patient with prominent shortening of the ribs and extremities, evident in the fetal period^24^. This variant was labeled VUS by InterVar and as DM in HGMD; it was included in set 4. The carrier frequencies of *DYNC2H1* were 0.00113 in set 1, 0.00225 in set 2, and 0.00337 in set 4 (Table 2).

#### *WDR34* (MIM# 615633)

A splicing variant, c.1372 + 2 T > C, was identified as heterozygous in one individual. This variant was not reported in ClinVar or HGMD but was interpreted as P by InterVar and classified in set 2 (Table 3). The estimated carrier frequency was 0.00028 (Table 2).

#### *IFT80* (MIM# 611263)

A stopgain variant, p.Ser134*, found in seven heterozygous individuals; two splicing variants, c.371-1 G > C, found in six heterozygous individuals and c.40-1 T > C, found in one heterozygous individual were classified as P by InterVar but were not reported in ClinVar or HGMD (Table 3). The carrier frequency was 0.00393 in set 2 (Table 2).

#### *IFT172* (MIM# 615630)

All five variants of *IFT172* result in a loss of function: p.Arg271* results in a stopgain variant; c.3229-1 G > C, c.2116-1 G > A, and c.571-1 G > A are splicing variants; and p.Thr663fs results in a frameshift variant. InterVar interpreted p.Arg271*, c.3229-1 G > C, c.2116-1 G > A, and c.571-1 G > A as P and p.Thr663fs as LP. p.Arg271* was included in set 1, and the remaining variants were included in set 2 (Table 3). One individual was heterozygous for all five variants, and the estimated carrier frequencies were 0.00028 in set 1 and 0.00141 in set 2 (Table 2).

#### *IFT140* (MIM# 266920)

Two stopgain variants, p.Gln998* and p.Arg1072*, one frameshift variant, p.Tyr923fs, and two synonymous SNVs, p.Thr1394Thr and p.Gly163Gly, were identified. None of these variants are reported in ClinVar. In InterVar, p.Gln998* was interpreted as P and p.Arg1072* and p.Tyr923fs were interpreted as LP, and thus included in set 2. Two variants, p.Thr1394Thr and p.Gly163Gly, were reported as DM in HGMD but not in InterVar and ClinVar; thus, they were included in set 4 (Table 3). All variants were found in one heterozygous individual each and the carrier frequencies were 0.00085 in set 2 and 0.00141 in set 4 (Table 2).

#### *WDR19* (MIM# 614376)

One frameshift variant, p.Leu211fs, and two splicing variants, c.2165 + 1 G > T and c.2782-2 A > G, were interpreted as P, and one nonsynonymous SNV, p.Gly789Arg, was identified as LP by InterVar. Only p.Leu211fs was included in set 1 and the remaining three pathogenic variants were included in set 2 (Table 3). The carrier frequencies were 0.00056 in set 1 and 0.00169 in set 2 (Table 2).

#### *TTC21B* (MIM# 613819)

A stopgain variant, p.Glu371*, and a splicing variant, c.430-2 A > C, were interpreted as P, and two frameshift variants, p.Tyr1282fs and p.Val1075fs, were interpreted as LP by InterVar. None of these four variants are reported in ClinVar or HGMD and all were included in set 2. Two nonsynonymous SNVs, p.Arg867Cys and pHis566Arg, were interpreted as VUS by InterVar but were included in set 4 because of their DM in HGMD (Table 3). The estimated carrier frequencies were 0.00141 in set 2 and 0.00197 in set 4 (Table 2).

The carrier frequencies, estimated from the sum of the allele frequencies, of the variants of the six ATD-associated genes were 0.00197 (set 1), 0.01182 (set 2), and 0.01408 (set 4). The proportions of potential patients calculated were 1/601,010 (set 1), 1/35,498 (set 2), and 1/26,553 (set 4) (Table 4).

## Discussion

We investigated variants of genes related to bone dysplasia using 3.5KJPNv2, which contains genomic information from a large general population. This is the first investigation of this type. We tried to estimate the allele frequency, carrier frequency, and proportion of potential patients in the general population by evaluating the pathogenic significance by original variant interpretation^13^. However, an automatic and efficient method for variant interpretation has not yet been established. Although many studies refer to existing databases such as ClinVar and HGMD for variant interpretation, the assessment of pathogenic significance in these databases is heterogeneous, with some reports misclassified as false-positive variants^25–27^. By combining useful bioinformatic tools such as InterVar with databases such as ClinVar and HGMD, we detected and evaluated not only previously reported but also novel variants and further classified the detected variants from sets 1–4 based on the reliability of pathogenic significance (Fig. 1).

We used a similar method as a previous study^13^, to estimate carrier frequency for diseases subject to neonatal screening. The proportions of potential patients with OI, ATD, and EvC based on pathogenic variants in set 2 were close to the reported incidence rates in Japanese patients^9^. Furthermore, the proportions of potential patients with HPP based on set 1 variants were close to the reported incidence rates in Japanese patients (Table 4).

Several factors are considered to be the reason for the difference between the estimated proportion of potential patients and the frequency in the previous report^9^. They might include variants with penetrance of <100%, those with false-positive results and those that are not present or have a mild effect on onset because allele frequencies are estimated from genomic information. It is possible that the carrier frequencies and the proportions of potential patients were overestimated, because they were considered to be pathogenically significant despite not being reported previously by the automatic determination of InterVar. On the other hand, some of the pathogenic variants identified as VUS by InterVar may actually be highly pathogenically significant. Furthermore, genetic testing is rarely performed in clinical practice, because many cases of bone dysplasia show severe clinical findings from the fetal stage^28^, resulting in abortion and fetal death^29^. Thus, the number of reported variants may be less than the actual number. For these reasons, the carrier frequencies and the proportions of potential patients may have been underestimated. In addition, we assume that the following factors could alter variant assessments and affect estimates of the carrier frequency and proportion of potential patients: (1) increasing the sample size of the whole-genome reference panel beyond the 3552 individuals and expanding the number of subjects analyzed in this study; (2) revising the ACMG-AMP variant interpretation guidelines; and (3) increasing the reported disease-associated variants and the number of causative variants enrolled in ClinVar and HGMD.

Few studies have compared bone dysplasia incidence between Japanese and other ethnic groups or reported bone dysplasia-associated variants unique to the Japanese population. Two pathogenic variants, p.Phe327Leu and c.1559delT, are frequent mutations in the *ALPL* gene in Japanese patients with HPP^19,30^. On the other hand, in Caucasians, there are two common variants: c.571 G > A, which is observed in 50% of mild HPP cases, and c.1133 A > T, which is the most common variant in perinatal benign HPP cases^31^. These variants were not detected in 3.5KJPNv2, suggesting ethnic differences in bone dysplasia-associated variants. In the future, with the expansion of whole-genome reference panels, variants with a high frequency in Caucasians and those that are potentially pathogenic will be detected.

This study has some limitations. First, the genomic information used here was based on the whole-genome reference panel data from 3552 individuals and the number of samples may not have been sufficient to obtain reliable estimates of the variant frequencies for rare diseases. However, it is possible to estimate the proportion of potential patients with autosomal recessive inheritance, making this the first study meaningful for autosomal recessive bone dysplasia in the Japanese population. Second, this study did not use the ten criteria in the ACMG-AMP variant interpretation guidelines that are not subject to the automatic determinations of InterVar. The reliability of pathogenic significance in the variants obtained in this study may be changed by adding evidence and evaluating them by custom analysis of InterVar. Third, large insertions/deletions and structural polymorphisms were not included in the public version of 3.5KJPNv2. That is, 3.5KJPNv2 was constructed by sequence analysis of short reads, but large insertions/deletions and structural polymorphisms may be detected by reanalysis using long-read NGS. Finally, the study looked at genes associated with bone dysplasia that are inherited in an autosomal recessive mode, including those with autosomal dominant or X-linked inheritance (OI and HPP). Investigating genomic information, including these modes of inheritance, may lead to more accurate estimates of carrier frequency and the proportion of potential patients.

It is difficult to appropriately estimate the carrier frequency and the proportion of potential patients based on pathogenic variants detected from genomic information. Furthermore, the establishment of an ideal and plausible method remains challenging. However, this is an unprecedented study of rare autosomal recessive bone dysplasias and the first study to attempt to estimate the carrier frequency and the proportion of potential patients from the allele frequency of pathogenic variants. From a genetic epidemiologic perspective, the findings from this study will help in the understanding of what types of pathogenic variants of bone dysplasia exist in the general population and the differences between reported frequencies observed in clinical practices in Japan and the proportions of potential patients calculated from allele frequencies. In terms of perinatal care, the findings of this study are expected to be useful for clinical diagnosis in cases where differential diagnosis is difficult, for accurate risk calculation such as the risk of recurrence for the next child, for information for parents who may be carriers, for genetic counseling such as prenatal diagnosis, and for personalized medicine such as neonatal treatment by utilizing variant information.

## Supplementary information

Table S1

## References

[1] Krakow D, Rimoin DL (2010). The skeletal dysplasias. Genet. Med..

[2] Forlino A, Marini JC (2016). Osteogenesis imperfecta. Lancet.

[3] Ozono K (1996). Identification of novel missense mutations (Phe310Leu and Gly439Arg) in a neonatal case of hypophosphatasia. J. Clin. Endocrinol. Metab..

[4] Camera G, Mastroiacovo P (1982). Birth prevalence of skeletal dysplasias in the Italian Multicentric Monitoring System for Birth Defects. Prog. Clin. Biol. Res..

[5] Orioli IM, Castilla EE, Barbosa-Neto JG (1986). The birth prevalence rates for the skeletal dysplasias. J. Med. Genet..

[6] Bonafe L (2015). Nosology and classification of genetic skeletal disorders: 2015 revision. Am. J. Med. Genet. A.

[7] Bae JS (2016). Comprehensive genetic exploration of skeletal dysplasia using targeted exome sequencing. Genet. Med..

[8] Taillandier A (2015). Molecular diagnosis of hypophosphatasia and differential diagnosis by targeted next generation sequencing. Mol. Genet. Metab..

[9] Satoh N, Murotsuki J, Sawai H (2009). Incidence rates of fetal bone dysplasias in Japanese (in Japanese). J. Jpn. Soc. Perin. Neon. Med..

[10] Tadaka S (2019). 3.5KJPNv2: an allele frequency panel of 3552 Japanese individuals including the X chromosome. Hum. Genome Var..

[11] Kuriyama S (2016). The Tohoku Medical Megabank Project: design and mission. J. Epidemiol..

[12] Kato, H., Kitano, T. & Iba, K. (eds). Surveillance registry for bone dysplasias of Japanese Orthopedic Association. p 20–26. (The Japanese Orthopaedic Association, Japan, 2016).

[13] Yamaguchi-Kabata Y (2019). Estimating carrier frequencies of newborn screening disorders using a whole-genome reference panel of 3552 Japanese individuals. Hum. Genet..

[14] Wang K, Li M, Hakonarson H (2010). ANNOVAR: functional annotation of genetic variants from high-throughput sequencing data. Nucleic Acids Res..

[15] Li Q, Wang K (2017). InterVar: clinical interpretation of genetic variants by the 2015 ACMG-AMP guidelines. Am. J. Hum. Genet..

[16] Landrum MJ (2016). ClinVar: public archive of interpretations of clinically relevant variants. Nucleic Acids Res..

[17] Stenson PD (2003). Human Gene Mutation Database (HGMD): 2003 update. Hum. Mutat..

[18] Richards S (2015). Standards and guidelines for the interpretation of sequence variants: a joint consensus recommendation of the American College of Medical Genetics and Genomics and the Association for Molecular Pathology. Genet. Med.

[19] Michigami T (2005). Common mutations F310L and T1559del in the tissue-nonspecific alkaline phosphatase gene are related to distinct phenotypes in Japanese patients with hypophosphatasia. Eur. J. Pediatr..

[20] Taketani T (2014). Clinical and genetic aspects of hypophosphatasia in Japanese patients. Arch. Dis. Child.

[21] Ren Y (2017). [Clinical analysis of 21 cases with short fetal femur in the third trimester]. Zhonghua Fu Chan Ke Za Zhi.

[22] Ruiz-Perez VL (2003). Mutations in two nonhomologous genes in a head-to-head configuration cause Ellis-van Creveld syndrome. Am. J. Hum. Genet..

[23] Zhang Z (2012). L. Identification of one novel mutation in the EVC2 gene in a Chinese family with Ellis-van Creveld syndrome. Gene.

[24] Okamoto T (2015). Novel compound heterozygous mutations in DYNC2H1 in a patient with severe short-rib polydactyly syndrome type III phenotype. Congenit. Anom..

[25] Dorschner MO (2013). Actionable, pathogenic incidental findings in 1,000 participants’ exomes. Am. J. Hum. Genet..

[26] MacArthur DG (2014). Guidelines for investigating causality of sequence variants in human disease. Nature.

[27] Shi L (2016). Long-read sequencing and de novo assembly of a Chinese genome. Nat. Commun..

[28] Ikenoue S (2018). Discordant fetal phenotype of hypophosphatasia in two siblings. Am. J. Med. Genet. A.

[29] Oberklaid F, Danks DM, Mayne V, Campbell P (1977). Asphyxiating thoracic dysplasia. Clinical, radiological, and pathological information on 10 patients. Arch. Dis. Child.

[30] Watanabe A (2011). Prevalence of c.1559delT in ALPL, a common mutation resulting in the perinatal (lethal) form of hypophosphatasia in Japanese and effects of the mutation on heterozygous carriers. J. Hum. Genet..

[31] Mornet E (2008). Hypophosphatasia. Best. Pr. Res Clin. Rheumatol..

